# Short and sweet: How glycans impact prion conversion, cofactor interactions, and cross-species transmission

**DOI:** 10.1371/journal.ppat.1009123

**Published:** 2021-01-14

**Authors:** Patricia Aguilar-Calvo, Julia A. Callender, Christina J. Sigurdson

**Affiliations:** 1 Department of Pathology, University of California, San Diego, La Jolla, California, United States of America; 2 Department of Medicine, University of California, San Diego, La Jolla, California, United States of America; 3 Department of Pathology, Microbiology, and Immunology, University of California, Davis, Davis, California, United States of America; University of Calgary, CANADA

## Introduction

Prion diseases are transmissible and incurable neurodegenerative disorders of humans and animals. Similar to other neurodegenerative disorders, such as Alzheimer’s disease and Parkinson’s disease, prion diseases are characterized by protein aggregation in the central nervous system (CNS). The key molecular event in prion disease is the conformational conversion of the cellular prion protein, PrP^C^, into a misfolded and aggregated conformer, PrP^Sc^, which templates further PrP^C^ misfolding [1]. Although this same protein misfolding event occurs in all prion diseases, affected individuals expressing identical PrP^C^ sequences can exhibit strikingly heterogeneous clinical and pathological phenotypes. These phenotypic differences have been linked to distinct PrP^Sc^ conformations, known as strains, yet the source of prion strain diversity is incompletely understood.

One potential contributor to strain diversity lies in the posttranslational modifications (PTMs) on PrP^C^, which add a layer of structural complexity to an otherwise highly conserved protein. PrP^C^ is posttranslationally modified by the covalent linkage of (i) 0 to 2 N-linked glycans at positions 181 and 197 (human PrP) and (ii) a glycosylphosphatidylinositol (GPI) moiety, which anchors PrP^C^ in the outer leaflet of the plasma membrane [2,3]. The N-linked glycans on PrP^C^ are branched [bi- (51%), tri- (32%), or tetra-antennary (17%)] and terminally sialylated, predominantly via alpha 2,6 linkages [4–6].

While necessary for regulating protein interactions and function, PTMs can profoundly modulate the pathogenesis of neurodegenerative diseases. For example, aberrant hyperphosphorylation of tau protein leads to tau detachment from microtubules and fibrillization into neurofibrillary tangles, a pathologic hallmark of Alzheimer’s disease. Prion diseases are no different, and the presence of PrP PTMs can markedly alter both the disease phenotype and transmission barrier. Here we explore how PrP PTMs impact prion conversion, cross-species transmission, the neuroinflammatory response, and PrP interaction with cofactors.

## How do PrP PTMs affect prion aggregate assembly, spread, and cell tropism?

The GPI anchor on PrP has a particularly profound impact on prion pathogenesis. In a landmark study, transgenic mice engineered to express GPI-anchorless PrP^C^ were inoculated with infectious prions [7]. Instead of the typical diffuse parenchymal deposits in the brain, the mice developed fibrillar extracellular amyloid exclusively in and around blood vessels similar to cerebral amyloid angiopathy, suggesting that removing the cellular tether enables PrP to transit through the interstitial fluid and subsequently assemble into perivascular fibrils. Moreover, GPI-anchorless prions circulated at high concentrations in the blood and accumulated systemically with an unusually broad tissue distribution that included adipose tissue, colon, and heart, even leading to a restrictive amyloid cardiomyopathy similar to certain systemic amyloidoses (transthyretin or immunoglobulin light chain amyloidosis) [8,9]. Interestingly, despite the high circulating prion titers, these fibril-forming prions spread poorly from peripheral entry sites into the CNS, quite unlike the rapid CNS entry of their GPI-anchored counterparts [10]. These observations suggest that the GPI anchor on PrP may limit fibril elongation and aggregate size and thereby impact the efficiency of spread through the peripheral nervous system and CNS, potentially occurring by axonal transport.

An unresolved question is how do GPI-anchorless prions cause neurodegeneration in the absence of membrane-bound PrP^C^? Although neuronal PrP^C^ expression is required for prion-induced neurotoxicity [11], GPI-anchorless PrP-expressing mice succumb to GPI-anchorless prion infection with approximately the same incubation period as wild-type mice [12]. This finding intriguingly illustrates that membrane-anchored PrP^C^ is not required for prion-induced neurodegeneration. Whether neurotoxic signaling occurs through PrP–cell membrane protein interactions extracellularly in trans, or intracellularly, or whether another mechanism underlies the toxicity remains to be determined.

Compared with animal models, patients developing Gerstmann–Sträussler–Scheinker disease (GSS) due to nonsense mutations in *PRNP*, for example, those encoding Q145X, Q160X, or Q163X develop GPI-anchorless fibrils that assemble as parenchymal amyloid plaques, vascular amyloid, or both [13]. These GSS cases show mild to no associated spongiform change in the brain [13,14], possibly due to exclusively extracellular prion conversion. Typically, the age of onset is young (third to fourth decade), and the disease course is prolonged (greater than 3 years) [13]. In contrast, genetic prion disease caused by GPI-anchorless near full-length PrP^Sc^ (Q226X) [15] is characterized by a rapid disease course and vascular amyloid, similar to the rapid course and vascular amyloid observed in the prion-infected GPI-anchorless PrP-expressing mice [12], illustrating how the length of the aggregating protein impacts the disease course and cellular targets.

**Fig 1:**
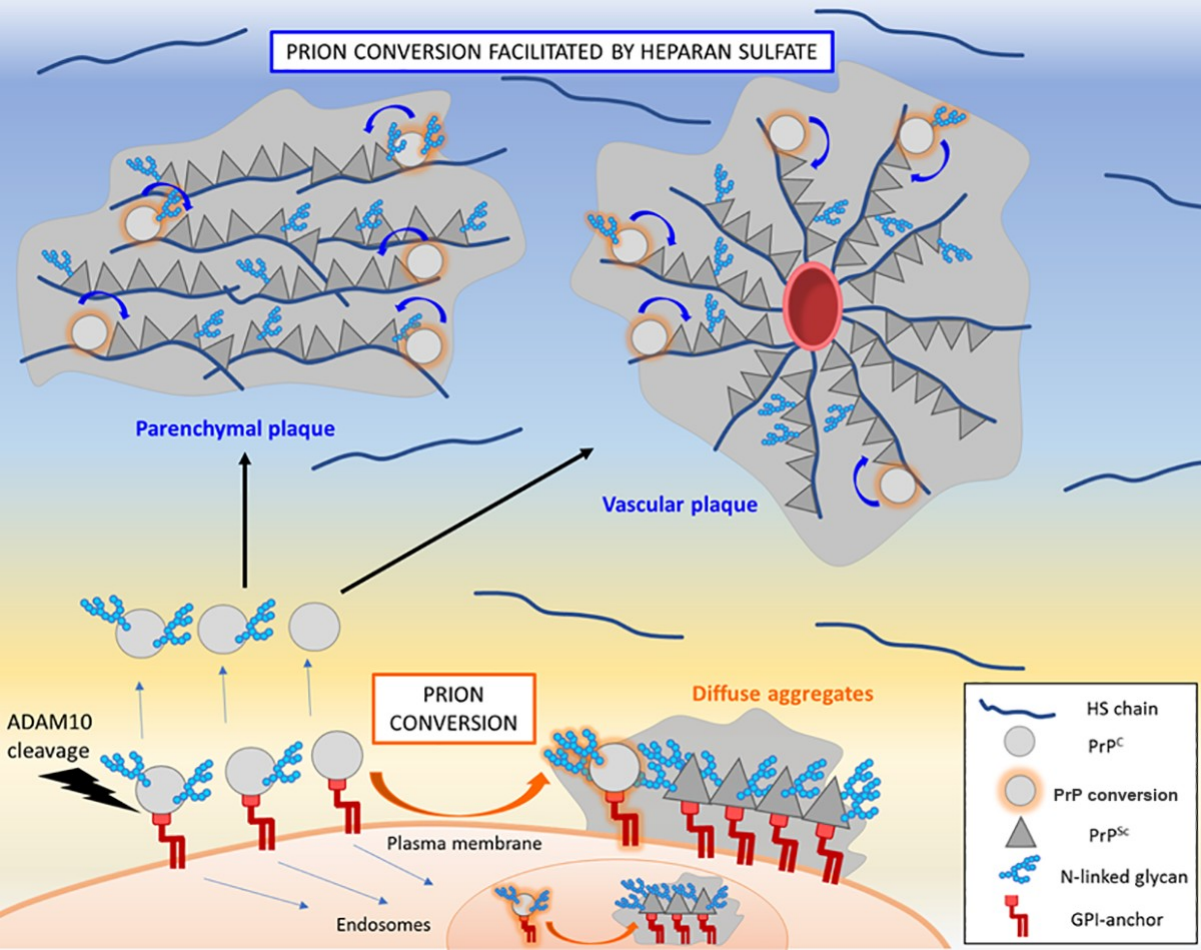
Model of how PrP PTMs may impact HS-mediated prion conversion. Prion conversion of *GPI-anchored prions* occurs at the cellular membrane or intracellularly within the endolysosomal pathway. *GPI-anchored prions* bind inefficiently to HS and form diffuse and small plaque-like aggregates containing a mixture of di-, mono-, and unglycosylated PrP^Sc^. Prion conversion of GPI-anchorless prions may occur extracellularly, as approximately 10% to 15% of the cellular prion protein (PrP^C^) is constitutively cleaved from the plasma membrane by ADAM10 [40]. Shed, ADAM10-cleaved PrP^C^ traffics through the interstitial fluid by bulk flow and binds HS chains in the extracellular matrix and vascular basement membrane. PrP^Sc^ also binds HS; thus, HS potentially facilitates PrP^C^ and PrP^Sc^ interaction and scaffolds assembly (curved arrows). Poorly glycosylated PrP (mono- and unglycosylated) binds to HS and is enriched in prion fibrils. ADAM10, A-disintegrin-and-metalloproteinase domain-containing protein 10; GPI, glycosylphosphatidylinositol; HS, heparan sulfate; PrP, prion protein; PrP^C^, cellular prion protein; PrP^Sc^, misfolded and aggregated prion protein.

Extensive study of the PrP glycans has revealed that the glycans can also markedly impact prion assembly pathways and the disease phenotype. Interestingly, abolishing both glycan attachment sites (through altering the PrP amino acid sequence) increases (i) spongiform degeneration, possibly due to intracellular conversion of unglycosylated, GPI-anchored PrP, and (ii) parenchymal plaque formation (for prions that convert A-disintegrin-and-metalloproteinase domain-containing protein 10 (ADAM10)-cleaved PrP, as 10% to 15% of PrP^C^ is ADAM10-cleaved) [16] (Fig 1). In contrast, the addition of a glycan instead promotes oligomer formation; even plaque-forming prions switch to predominantly oligomeric prions and show a profound increase in prion spread from extraneural organs into the CNS [16,17]. Thus, by eliminating *either* the GPI-anchor *or* the N-linked glycan attachment sites, PrP shows an increased tendency to assemble into fibrillar plaques. Establishing how the glycans and GPI anchor impact particle size and influence entry and spread into and through the CNS would be an interesting avenue for future studies.

## How do PrP PTMs impact neuroinflammation in prion disease?

Terminal sialic acid residues on cell surface proteins and lipids contribute to a healthy mammalian sialoglycocalyx, enabling the immune system to differentiate “self” from “nonself.” Apoptotic or aging cells experience a decrease in the sialic acid content of the glycocalyx, leading to the activation of microglia and macrophages [18]. As an extracellular glycoprotein, PrP—with terminal sialic acid residues on N-linked glycans—is particularly well-suited to activate microglia. In this vein, Srivastava and colleagues showed that PrP^Sc^ purified from prion-infected brain triggers a pro-inflammatory response when added to cultured BV2 microglial cells, and this response was even stronger when adding PrP^Sc^ lacking terminal sialic acids [19].

*In vivo* studies have shown that the astrocytic response to prion infection varies by brain region and *does not* strongly correlate with PrP^Sc^ deposition; however, reactive microgliosis *does* correlate with PrP^Sc^ deposition in mice [20] and humans [(for type 1 sporadic Creutzfeldt–Jakob disease (sCJD)] [21]. Of note, PrP^Sc^ sialylation pattern and levels vary by brain region and are controlled by expression of various sialyltransferase enzymes [22]. Lower sialylation status of PrP^Sc^ may therefore be at least partially responsible for instigating more pro-inflammatory responses from microglia in brain regions with lower sialyltransferase expression [22]. Collectively, these studies support a testable hypothesis in which lower levels of sialylated PrP^Sc^ further activate neuroinflammatory cascades and may provide an explanation for the more severe reactive microgliosis observed with type 1 versus type 2 sCJD [21].

## How do PrP PTMs impact prion conversion and cross-species prion transmission?

Prion conversion requires the interaction of PrP^Sc^ with PrP^C^ for template-assisted misfolding, which is modulated by (i) host–recipient PrP amino acid sequence similarity and (ii) the prion strain. PTMs on PrP^C^ are not required for prion conversion, as shown by prion infection of mice expressing GPI-anchorless or unglycosylated PrP^C^ [7,23]. However, PrP^C^ glycosylation can impact conversion in a conformation-dependent manner. For example, *in vitro*, human sCJD prions (subtypes 129MM1 and MM2) most efficiently seed unglycosylated PrP^C^ substrates, whereas variant CJD (vCJD) prions preferentially seed glycosylated PrP^C^ substrates [24], illustrating the profound effect of the PrP^Sc^ fold on PrP^C^ glycoform recruitment.

PrP PTMs also modulate cross-species prion transmission. For example, the absence of an N-linked glycan at residue 196 enabled the transmission of human sCJD (subtype MM2) to transgenic mice [25], overcoming a well-established human–mouse species barrier [26]. Similarly, the absence of the GPI anchor enabled transmission of mouse prions to human PrP-expressing mice [27]. This finding is particularly intriguing in light of only subtle conformational differences between the GPI-anchored and GPI-anchorless versions of the same strain [28]. Although early studies identified the N-linked glycans as imposing spatial and electrostatic constraints to the PrP^C^—PrP^Sc^ interaction, more recent work directly showed a major role for the terminal sialic acids; some prion strains converted less sialylated PrP^C^ glycoforms, whereas other strains converted a diverse array of sialylated glycoforms [29]. These studies reveal a conformation-specific effect of the negatively charged terminal sialic acids on prion conversion, including cross-species transmission [29,30].

## How do PrP PTMs impact interaction with cofactors?

Cofactors, including RNA, lipids, and polyanions, promote *in vitro* prion conversion, yet they are not essential for inducing conversion of recombinant PrP^C^ into infectious PrP^Sc^ [31–33]. However, the prion conformation, PrP PTMs, and specific cofactors are tightly intertwined; recent data shows that the conformation of the PrP^Sc^ “seed” determines the cofactor and PrP^C^ glycoform preferences [34]. We recently found that heparan sulfate (HS), an abundant polyanion in the brain, accelerates prion disease by enhancing the conversion and assembly of extracellular, ADAM10-cleaved PrP into parenchymal plaques [35] (Fig 1). Interestingly, the glycans impair PrP binding to heparin (a sulfated glycosaminoglycan varied), possibly due to electrostatic repulsion between the anionic glycans and the sulfate groups or by masking a heparin binding domain [36]; *di*-glycosylated PrP^C^ shows the *lowest* heparin binding affinity [16]. Consistent with this finding, in mice, *di-*glycosylated PrP^Sc^ showed the least HS bound, whereas unglycosylated PrP^Sc^ showed the most HS bound [35]. Additionally, GPI-anchorless prion fibrils, which are poorly glycosylated, bind high levels of HS. Taken together, these results show how the PrP glycans can impede fibril formation, potentially through restricting PrP binding to anionic cofactors, including HS (Fig 1).

Collectively, these findings have relevance for both sporadic and genetic forms of prion disease and may help explain the geneses of mixed pathological phenotypes that include both diffuse PrP^Sc^ aggregates and plaques [37], for example, sCJD subtype MV2 cases develop synaptic and plaque-like deposits in the cerebral cortex and Kuru plaques in the cerebellum [38,39]. Given that 10% to 15% of PrP^C^ is carboxyl-terminally truncated just proximal to the GPI anchor [40], the assembly of GPI-anchorless PrP into HS-rich parenchymal plaques may be occurring in sporadic disease. Additionally, HS may also contribute to the assembly of GPI-anchorless PrP in genetic prion disease, as GSS cases develop HS-laden plaques [35]. Future studies that define the PrP sequences, PTMs, and cofactors bound to prion plaques in human brain may reveal further insights into the nature and formation of the complex mixed aggregate assemblies in prion disease.

In conclusion, prion-affected individuals vary widely in their clinical progression and affected brain regions, phenotypic traits that may be modulated by PrP PTMs. Thus, understanding how the PTMs influence PrP^Sc^ folding, assembly pathways, and cofactor interactions is important for tailoring therapies specific to the disease subtype, for example, by modulating ADAM10-cleavage or impairing prion binding to HS. Ultimately, determining how PrP PTMs impact interactions with endogenous cofactors has the potential to advance the rational design of anti-prion therapies.
